# A reproducible low-dimensional architecture of human white-matter connectivity

**DOI:** 10.21203/rs.3.rs-10637290/v1

**Published:** 2026-08-14

**Authors:** D. Tomasi, B. Elliott, J. Sarlls, N.D. Volkow

**Affiliations:** 1 National Institute on Alcohol Abuse and Alcoholism, National Institutes of Health, Bethesda, Maryland, USA.; 2 National Institute of Neurological Disorders and Stroke, National Institutes of Health, Bethesda, Maryland, USA.

## Abstract

The human brain is organized by large-scale functional gradients, but whether white-matter connectivity defines similarly reproducible and biologically interpretable axes of cortical, subcortical, and cerebellar organization remains unclear. Here, we introduce Structural Connectivity Gradient Mapping (SCGM), a framework for deriving low-dimensional structural connectivity gradients from diffusion MRI tractography using a whole-brain parcellation spanning 438 cortical, subcortical, and cerebellar regions. Across repeated scans, an independent onsite cohort, and the Human Connectome Project Young Adult cohort, SCGM-derived gradients were highly reproducible and robust across datasets. Four dominant gradients captured most structural-connectivity variance and mapped onto distinct anatomical axes: hemispheric laterality and geometry, dorsal-ventral organization, cingulate-temporal organization, and a cerebellar-transmodal axis. Despite their reliability, structural gradients showed only limited correspondence with functional connectivity gradients, indicating that white-matter architecture and functional organization capture partly distinct dimensions of brain organization. Age effects were strongest for higher-order structural gradients, particularly the cerebellar-transmodal axis, where older age was associated with reduced separation between cerebellar and transmodal cortical parcels. These findings identify a reproducible low-dimensional architecture of human white-matter connectivity, establish SCGM as a framework for mapping structural-connectivity gradients, and reveal age-related remodeling of a cerebellar-transmodal axis linking distributed cortical and cerebellar systems.

## Introduction

The human brain is organized by large-scale patterns of anatomical connectivity that constrain and enable distributed neural function^1^. White-matter pathways impose spatial, metabolic, and topological constraints on communication between neural systems, while long-range connections support integration among distant cortical, subcortical, and cerebellar territories^2^. Traditional connectome analyses have characterized this architecture using discrete networks, graph-theoretic measures, or tract-specific reconstructions^1,3^. These approaches have been highly informative, but they may obscure continuous axes of organization that cut across conventional network boundaries. Gradient methods address this limitation by embedding high-dimensional connectivity profiles into low-dimensional coordinate systems, revealing smooth transitions in brain organization rather than assigning each region to a single module^4^. This framework has become influential in functional, microstructural, and multimodal neuroimaging because it provides a compact description of macroscale brain organization across multiple spatial scales.

Functional-gradient studies have shown that cortical organization can be described by reproducible axes of connectivity variation, including the canonical sensory-transmodal hierarchy and additional gradients related to visual, somatomotor, attentional, and association-system organization^4–7^. However, whether structural connectivity (SC) provides an analogous low-dimensional coordinate system remains less clear. Structural connectivity differs from functional connectivity (FC) in important ways: it is constrained by physical distance, hemispheric layout, tract geometry, and the limitations of diffusion MRI tractography^3,8–10^. At the same time, it provides the anatomical substrate through which functional interactions are shaped^2,11–13^. Long-distance anatomical connections are metabolically and spatially costly, but they allow distant systems to exchange diverse inputs and support complex dynamics^14,15^. Low-dimensional embeddings of structural connectivity may therefore reveal biologically meaningful axes of white-matter organization that are not reducible to spatial proximity alone^15^.

A key unresolved question is whether structural gradients recapitulate the axes observed in functional-gradient studies or instead reveal distinct anatomical principles. This distinction is important because structure-function coupling is not uniform across the brain. Some functional topographies may be closely scaffolded by structural connectivity, whereas others may depend more strongly on polysynaptic communication, shared inputs, neuromodulatory state, or dynamic functional coupling^11,16,17^. Whole-brain structural gradients may capture anatomical patterns that are underrepresented in cortex-only functional analyses, including hemispheric asymmetries^18^, cingulate-temporal association pathways^19^, and cortico-cerebellar organization^20^. The cerebellum is especially relevant in this context because it participates in distributed motor, cognitive, and association circuits rather than serving only motor control^21^. Intrinsic connectivity studies have shown that cerebellar territories map onto multiple cerebral networks^20^, and cerebellar gradients span motor-to-association organization^22^.

Reproducibility is a central challenge for gradient-based connectomics. Embedding solutions can vary in component order and sign^6,23^, and diffusion MRI estimates of structural connectivity are sensitive to angular resolution, tractography choices, and acquisition conditions^3,12,13,24^. For structural gradients to be useful as quantitative neurobiological phenotypes, they must be stable across repeated scans, robust across datasets, and reproducible under alternative reconstruction choices. This is particularly important for studies aiming to relate gradients to individual differences, aging, or disease. Establishing reproducibility is therefore a prerequisite for interpreting structural gradients as meaningful axes of brain organization rather than dataset-specific embeddings.

Structural gradients may also provide a sensitive framework for studying age-related variation in brain connectivity. Aging affects white-matter microstructure and structural network organization, with particular relevance for long-range association and cerebello-cortical pathways^25^. Cerebellar aging has been linked to motor and cognitive decline^26,27^, and diffusion MRI studies implicate cerebellar and cerebello-thalamo-cortical white matter in age-related cognitive variation^28,29^. These observations motivate the possibility that higher-order structural gradients, especially those involving cerebellar and association-system connectivity, may show age-related remodeling.

Here, we introduce Structural Connectivity Gradient Mapping (SCGM), a framework for resolving the principal low-dimensional axes of whole-brain white-matter connectivity, and test whether these gradients provide a reproducible and biologically interpretable coordinate system for human brain organization. Using diffusion MRI tractography and a cortical, subcortical, and cerebellar parcellation, we characterize the anatomical organization of SCGM-derived gradients and assess their test-retest reliability, cross-dataset reproducibility, correspondence with functional connectivity gradients, sensitivity to age, and robustness to alternative tractography reconstruction parameters. We identify separable hemispheric, dorsal-ventral, cingulate-temporal, and cerebellar-transmodal axes that are highly reproducible across scans and datasets. Together, these findings establish SCGM as a generalizable framework for mapping low-dimensional white-matter connectivity architecture and reveal age-related remodeling of a cerebellar-transmodal axis linking distributed cortical and cerebellar systems.

## Results

### Low-dimensional structural connectivity gradients

To characterize the low-dimensional organization of human structural connectivity, we applied a gradient-based framework to multimodal MRI data from 61 healthy adults (Fig. 1). Resting-state fMRI was used to derive functional connectivity matrices and functional gradients, whereas diffusion MRI tractography with SIFT2 streamline weighting^30^ was used to derive structural connectivity matrices and structural gradients within the same iCSC-438 parcellation^31^. This common parcellation enabled direct parcel-wise comparison between structural and functional gradients while preserving modality-specific connectivity information. We first estimated diffusion map embeddings from the group-mean structural connectome and then tested whether the resulting structural gradients defined reproducible, anatomically interpretable axes of brain organization, whether they corresponded to functional gradients beyond gross spatial geometry, and whether they captured variation associated with age and sex.

Weighted-degree analysis of the group-mean structural connectivity matrix revealed heterogeneous connectivity across tract-defined endpoint regions (Fig. 2a). Structural connectivity strength was concentrated in posterior parietal, medial parietal, early visual, temporal pole, premotor, and prefrontal association parcels, indicating that high structural connectivity was localized to a subset of sensory-associated and transmodal territories rather than distributed uniformly across the brain. At the network level, most network pairs showed weak mean structural connectivity, whereas a smaller subset showed relatively high connectivity, consistent with selective anatomical coupling among systems (Fig. 2b). Superior longitudinal/arcuate fasciculus endpoints^32–34^ were dominated by dorsal attention, somatomotor, and frontoparietal/control regions, whereas inferior longitudinal, inferior fronto-occipital, and uncinate fasciculus endpoints^35–37^ emphasized visual, ventral temporal, orbitofrontal, and default/transmodal regions (Supplementary Table S1). Structural connectivity matrices were highly reproducible across repeated acquisitions (R^2^=0.94±0.02; Supplementary Fig. S1).

Diffusion map embedding identified four dominant structural connectivity gradients that together accounted for 68% of the variance among the first 10 gradients (Figs. 2c, 3, and S2–S5). These gradients captured distinct anatomical axes: G1 reflected hemispheric laterality and spatial geometry, G2 a dorsal-ventral structural axis, G3 a cingulate-temporal axis, and G4 a cerebellar-transmodal axis.

### Anatomical interpretation of structural gradients

G1 was dominated by hemispheric laterality but also showed spatially structured variation. At the group level, Cartesian coordinates alone explained 19.9% of parcel-wise G1 variance, whereas hemisphere alone explained 62.8%. Adding hemisphere to the coordinate-only model markedly improved fit (ΔR^2^=0.60), and the best-fitting model included coordinate-by-hemisphere interactions (R^2^=0.93), indicating that the spatial expression of G1 differed across hemispheres. Across scans, hemisphere alone explained 64±11% of G1 variance, coordinates alone explained 12±7%, and the interaction model explained 88±3%, confirming that G1 captured a reproducible laterality axis with additional geometry-dependent structure.

G2 reflected a dorsal-ventral structural organization. At the group level, a tract-derived dorsal-ventral endpoint-loading contrast explained 78.2% of G2 variance, compared with 24.5% explained by Cartesian coordinates alone (Fig. 4a,b). Adding the dorsal-ventral contrast to the geometry-only model increased explained variance by ΔR^2^=0.61, yielding a combined model R^2^ of 0.85. Across subject-session scans, coordinates explained 42±10% of G2 variance, the dorsal-ventral contrast explained 46±13%, and the combined model explained 73±11%. Thus, G2 captured a reproducible dorsal-ventral structural connectivity axis that was not reducible to spatial position.

G3 reflected a broad medial-to-lateral structural-connectivity pattern whose opposing extrema were centered in the cingulate and lateral temporal cortices. The negative pole was strongest in the cingulate cortex and attenuated into the precuneus, whereas the positive pole reached its maximum in lateral temporal cortex, with weaker positive loadings extending into prefrontal and parietal regions. We therefore refer to G3 as a cingulate-temporal axis, using these regions to define its dominant endpoints. At the group level, the cingulate–temporal endpoint-loading contrast explained 83.9% of G3 variance, compared with 47.5% explained by Cartesian geometry (Fig. 4a,c). Adding the cingulate-temporal predictor to the geometry-only model substantially improved fit (ΔR^2^=0.37), whereas adding geometry to the cingulate–temporal model explained no additional variance. Across subject-session scans, both spatial coordinates and the cingulate–temporal contrast explained substantial G3 variance, and modeling the cingulate and temporal loadings separately further increased the explained variance to 63±10%. These findings support G3 as a reproducible cingulate–temporal structural-connectivity axis embedded within a broader medial-to-lateral cortical organization.

G4 was dominated by a cerebellar-transmodal axis. At the group level, cerebellar loading alone explained more G4 variance than Cartesian geometry (R^2^=0.44 versus R^2^=0.15), and a cerebellar-transmodal endpoint-loading contrast provided a stronger single-axis explanation of G4 (R^2^=0.53; Fig. 4a,d). Adding this contrast to the geometry-only model improved fit substantially (combined R^2^=0.60; ΔR^2^=0.46). Modeling cerebellar and transmodal loadings separately provided the best fit (R^2^=0.68), supporting G4 as a cerebellar-transmodal structural connectivity axis.

### Structural gradients show limited correspondence with functional gradients

We next compared structural gradients with independently derived functional gradients in the Discovery sample after component matching and sign alignment. The strongest significant correspondence among the first four gradients was between SC G2 and FC G4 across cortical parcels (r=0.445, p_spin_=0.0018). Other correspondences among the first four structural and functional gradients were weaker or not significant after spin correction. Thus, despite their high reproducibility, structural gradients showed limited correspondence with functional connectivity gradients, indicating that white-matter architecture and functional organization capture partly distinct dimensions of brain organization.

### Structural gradients are reproducible across sessions, cohorts, and parcellations

Test-retest analyses demonstrated excellent within-participant reproducibility of SC gradients between repeated sessions after template alignment (Fig. 5a,b). Across G1–G4, mean within-participant spatial correlations were high (0.91<r<0.97), and parcel-wise reliability was similarly strong (ICC(3,1)>0.83; Fig. 5c). Gradient strength and eigenvalues did not differ systematically between sessions, indicating that the SC gradient architecture was stable across repeated acquisitions.

The gradient architecture was also reproduced in an independent onsite replication sample, with near-identical spatial correspondence between Discovery and Replication gradients across G1–G4 (0.99<r<1.00; p_spin_<0.0001; Fig. 6a,c). External validation in 100 individuals from the Human Connectome Project Young Adult cohort showed strong generalizability, with HCP-YA group-level SC gradients closely matching Discovery gradients after component and sign alignment (0.85<r<0.99; p_spin_<0.0012; Fig. 6b,d). Although the first two gradients were reordered in HCP-YA, their spatial topographies were preserved after alignment. These findings support the robustness of SC gradients across repeated scans, acquisition conditions, and independent cohorts.

Atlas-sensitivity analyses showed that the principal SC gradient architecture was broadly preserved when gradients were estimated using the AAL116 atlas (Supplementary Fig. S6). After surface projection, component matching, and sign alignment, AAL116-derived showed significant spatial correspondence with iCSC-438 gradients across G1–G3 (r>0.76; p_spin_<0.005). The first four of the 10 gradients together explained 60.5% of the variance, compared with 68% for the corresponding iCSC-438 solution, indicating that the leading structural architecture was preserved across parcellations but was distributed across a larger number of components in the coarser atlas. The correspondence for iCSC-438 G4 was weaker but remained significant with AAL116 component 10 (r=0.47, p_spin_=0.002), indicating that leading gradients were not specific to the primary parcellation, although higher-order gradients were more sensitive to atlas resolution and component ordering.

Sensitivity analyses using a more permissive FOD amplitude cutoff also supported the robustness of the principal structural architecture. Lowering the FOD cutoff from 0.10 to 0.03 preserved the major gradient topographies but altered their relative ordering (Supplementary Fig. S7). Specifically, FOD=0.10 G1, G2, and G4 matched FOD=0.03 G2, G1, and G3, respectively (|r|>0.84; p_spin_<0.0004). These findings indicate that component identity should be interpreted anatomically and by spatial correspondence rather than solely by gradient rank.

### Age-related remodeling of higher-order structural gradients

Parcel-wise linear mixed-effects analyses in the Discovery sample revealed significant age-related variation in SC gradients after template alignment. These effects remained robust after adjustment for intracranial volume, indicating that they were not attributable to global head-size differences. Adjustment for mean cortical thickness did not attenuate the age-related SC-gradient effects, and mean cortical thickness was not a significant predictor after correction. Thus, age-related variation in SC gradients was not explained by global cortical thinning, although contributions from regional atrophy, ventricular expansion, white-matter tissue loss, or free-water effects cannot be excluded.

Age effects were strongest for G4 and G3. For G4, 213 parcels showed nominal age associations, 173 survived within-gradient FDR correction, and 118 remained significant after FDR correction across all tested gradient-parcel combinations (Fig. 7 and Table S2). For G3, 67 parcels showed nominal age associations, 11 survived within-gradient FDR correction, and 17 survived correction across all tests. Age effects were more limited for G1 and G2, with no parcels surviving within-gradient FDR correction. Intracranial-volume effects were weaker than age effects overall, and corrected ICV associations were mainly observed for G3. Sex effects were modest, with only isolated corrected parcels in G1 and G3. These findings identify age as the dominant demographic contributor to SC-gradient variation, especially for higher-order gradients G3 and G4.

Older age was not significantly associated with mean or median cerebellar clustering coefficient after adjustment for sex and ICV but was associated with increased median graph distance between G4 cerebellar-pole parcels and transmodal parcels (p=0.008; q_FDR_=0.048, partial r=0.49). Median direct connectivity weight to transmodal parcels showed a consistent negative association with age, although this effect did not survive FDR correction (p=0.029; q_FDR_=0.089, partial r=−0.52). The age-related attenuation of the cerebellar-transmodal gradient was reproduced using the more permissive FOD=0.03 tractography pipeline (Supplementary Fig. S8). Age-related increases in graph distance were specific to the G4 cerebellar-transmodal axis. Among parcels showing age effects on G3, neither mean nor median graph distance varied significantly with age after correction for multiple comparisons. Instead, older age was associated with increased local clustering within the G3 parcel set (p=0.0006; q_FDR_=0.003, partial r=0.41).

To assess whether age-related G4 effects were accompanied by global cerebellar microstructural differences, we extracted FA and MD from subject-specific whole-cerebellum white matter. Mean FA was 0.39±0.03 and mean MD was 0.73±0.03. Neither measure showed a significant association with age after FDR correction (FA: p=0.053, partial r=−0.25; MD: p=0.80, partial r=0.03).

Motion-adjusted linear mixed-effects analyses of functional gradients revealed weaker demographic sensitivity than observed for structural gradients. Age effects were nominally present across FC gradients but did not survive correction across all gradient-parcel tests, and sex effects did not survive FDR correction for any FC gradient. By contrast, mean framewise displacement showed robust associations with FC gradients, especially FC G2. Thus, after controlling for head motion, FC gradients showed substantially weaker age and sex effects than SC gradients, whose age associations remained robust after ICV adjustment.

## Discussion

This study identifies a reproducible low-dimensional architecture of human structural connectivity and shows that its principal axes capture distinct anatomical patterns with limited correspondence to functional-gradient organization and selective sensitivity to age. Across group-level, individual, test-retest, onsite replication, and external HCP-YA analyses, structural connectivity gradients were highly stable, indicating that these axes reflect robust properties of brain structural connectivity rather than dataset-specific embedding solutions. The leading gradients mapped onto hemispheric, dorsal-ventral, cingulate-temporal, and cerebellar-transmodal axes. Structural gradients showed only limited correspondence with functional gradients, with the strongest structure-function relationship observed between the dorsal-ventral structural gradient and a non-leading functional gradient. Age-related variation was most pronounced for the cerebellar-transmodal gradient, where effects were concentrated in the cerebellar pole and were consistent with attenuation of this axis in older individuals. Together, these findings suggest that structural connectivity gradients provide a reliable anatomical coordinate system for human brain organization while revealing dimensions of white-matter architecture that are only partly expressed in functional-gradient space.

Gradient approaches have become influential because they move beyond discrete network labels to describe continuous axes of connectivity variation across the brain^39^. Functional-gradient studies have established reproducible cortical hierarchies, including the canonical sensory-to-transmodal axis, and microstructural-gradient studies have shown that cortical tissue properties are organized along related but non-identical macroscale dimensions^4,40,41^. Our findings extend this framework from cortical function and microstructure to whole-brain structural connectivity. Despite differences in acquisition conditions and diffusion sampling across datasets, the same principal structural-gradient architecture was recovered across repeated scans, an onsite replication cohort, and the HCP-YA cohort. This convergence supports the interpretation of structural gradients as stable anatomical phenotypes that can be compared across individuals and cohorts.

The leading structural gradients did not recapitulate a single sensory-to-association hierarchy. Instead, they captured multiple anatomical axes. G1 was dominated by hemispheric laterality and spatial geometry, suggesting that the strongest mode of variation in the tractography-derived structural connectome reflects the balance of intrahemispheric and interhemispheric connectivity^42^. Because diffusion tractography is sensitive to distance effects, crossing fibers, and reconstruction biases^3,12,13,24^, this laterality-dominated axis should be interpreted cautiously. It likely reflects both true hemispheric organization and methodological properties of tractography-based connectome reconstruction.

Subsequent gradients captured more specific anatomical dimensions. G2 mapped onto a dorsal-ventral structural axis and showed the strongest low-order correspondence with functional-gradient organization, although this relationship involved a non-leading functional gradient and other structure-function correspondences were weak. This pattern supports limited rather than global convergence between structural and functional embeddings. G3 reflected a cingulate-temporal axis, consistent with major association pathways linking medial frontal, cingulate, retrosplenial, parahippocampal, and temporal-limbic territories^19,43,44^. G4 was dominated by a cerebellar-transmodal axis, consistent with evidence that the cerebellum participates in distributed motor, cognitive, and association circuits rather than serving only motor control^20,22,45,46^. Across G2–G4, tract- and endpoint-loading models explained substantial variance beyond Cartesian coordinates, indicating that these gradients capture connectivity-specific anatomical organization rather than spatial smoothness alone.

The limited correspondence between structural and functional gradients has important implications for interpreting macroscale brain organization. White-matter connectivity provides the anatomical substrate for functional communication^47^, but structure-function coupling is spatially heterogeneous and shaped by polysynaptic communication, and dynamic coupling^48–50^. The present findings are consistent with this view. Structural gradients were highly reproducible, but they did not simply mirror the dominant functional gradients. Instead, they identified anatomical axes that were only selectively expressed in functional-gradient space. This suggests that structural and functional gradients provide complementary coordinate systems: one describing relatively stable anatomical constraints and the other describing dynamic patterns of functional coupling.

The age-related attenuation of the cerebellar pole of G4 highlights the sensitivity of higher-order structural gradients to biologically meaningful variation. Because G4 was dominated by a cerebellar negative pole, positive age effects within cerebellar parcels indicate that this pole became less negative with increasing age, consistent with compression of the cerebellar-transmodal structural axis. Graph analyses supported this interpretation: older age was associated with increased graph distance between cerebellar-pole parcels and transmodal cortical parcels, with a directionally consistent reduction in direct cerebellar-transmodal connectivity strength. These findings suggest that age-related G4 variation may reflect reduced topological integration between cerebellar and transmodal systems rather than altered local cerebellar clustering.

The cerebellar localization of age effects is notable because cerebellar aging has been linked to both motor and cognitive decline^26^, and diffusion MRI studies implicate cerebellar and cerebello-thalamo-cortical white matter in age-related cognitive variation^28,29^. In the present study, conventional tensor-derived FA and MD from whole-cerebellum white matter did not show significant age associations, suggesting that gradient and graph-distance measures may be more sensitive to distributed cerebellar-transmodal organization than global cerebellar white-matter summary measures. However, this interpretation remains cautious because cerebellar tractography is challenging and can be affected by thin white matter, partial-volume effects, susceptibility-related distortions, and reconstruction choices^13,51^.

Several limitations should be considered. Diffusion tractography provides an indirect estimate of structural connectivity and remains sensitive to false positives, false negatives, distance effects, crossing fibers, and preprocessing choices^13^. Although the Discovery and Replication datasets supported robust gradient recovery and the HCP-YA cohort provided external validation with higher angular diffusion sampling, endpoint-level interpretations should be treated cautiously. The cerebellar-transmodal gradient also depends on accurate parcellation and reconstruction of cerebellar pathways; future work should test its stability across cerebellum-specific parcellations and alternative tractography pipelines. In addition, spin-based null models are most straightforward for cortical surface analyses and are less well suited to subcortical and cerebellar parcels, so inference involving whole-brain gradients requires caution. Finally, the age analyses were observational and cross-sectional. Longitudinal lifespan datasets, combined with behavioral measures of motor and cognitive function, will be needed to determine whether attenuation of the cerebellar-transmodal axis tracks age-related change within individuals and predicts functional outcomes.

Beyond the specific organizational principles identified here, SCGM provides a general framework for studying low-dimensional structural connectome organization. By representing structural connectivity as continuous gradients rather than discrete modules or individual connections, SCGM enables direct characterization of spatially distributed white-matter architecture and facilitates comparisons across individuals, cohorts, and imaging modalities. The accompanying open-source pipeline is intended to support application and extension of SCGM in independent datasets.

In conclusion, structural connectivity gradients provide a reproducible and biologically interpretable coordinate system for structural brain connectivity. Rather than recapitulating a single functional hierarchy, the leading gradients captured multiple anatomical axes that were stable across sessions, cohorts, parcellations, and tractography reconstruction parameters, but showed only limited correspondence with functional gradients. The age-related attenuation of the cerebellar-transmodal gradient further suggests that higher-order structural gradients may index biologically meaningful remodeling of cortico-cerebellar systems. These findings position structural gradients as reliable quantitative phenotypes for studying anatomical organization, structure-function coupling, and age-related variation in human brain connectivity.

## Methods

### Participants

Eighty-nine healthy non-smoking adults aged 18 years or older were included. Due to changes in MRI hardware, participants were analyzed as two cohorts. The Discovery cohort included participants scanned between January and September 2016 (n=61; age, 43.3±13.7 years; 34 females), and the Replication cohort included participants scanned between March and December 2015 (n=28; age, 37.1±12.8 years; 10 females). Data analyzed in the present study were acquired under NIH protocol 14-AA-0144 (“Reliability of the Human Brain Connectome”; ClinicalTrials.gov NCT02193425). The present work constitutes a secondary analysis of neuroimaging data acquired under this protocol and does not report the registered primary trial outcome. The study was approved by the National Institutes of Health Combined Neurosciences White Panel Ethics Committee and was conducted in accordance with the Declaration of Helsinki. All participants provided written informed consent before participation. External validation was performed in an independent de-identified sample of 100 participants from the Human Connectome Project Young Adult cohort (HCP-YA; age, 29.0±3.5 years; 57 females), in accordance with the HCP data-use terms and the ethics approvals obtained by the HCP consortium.

### MRI data acquisition

Discovery and Replication participants were scanned in two sessions on separate days approximately two weeks apart using a 3.0 T Siemens Magnetom Prisma scanner equipped with either a 32-channel head coil in the Discovery cohort or a 20-channel head coil in the Replication cohort^52^. Participants rested with eyes open during both diffusion-weighted imaging (DWI) and resting-state fMRI.

For both cohorts, diffusion MRI was acquired using a 2D echo-planar spin-echo diffusion sequence with anterior-posterior phase encoding, 2.2 mm isotropic resolution, TR=7400 ms, TE=73 ms, flip angle=90°, GRAPPA in-plane acceleration factor=2, effective echo spacing=0.28 ms, total readout time=30.52 ms, and pixel bandwidth=2390 Hz/pixel. The diffusion protocol included 45 volumes across three nonzero diffusion shells and interspersed b0 images, with b-values of 0 s/mm^2^×3, 200 s/mm^2^×6, 500 s/mm^2^×6, and 1100 s/mm^2^×30. Reversed phase-encoding acquisitions were collected for susceptibility-distortion correction.

In the Discovery cohort, resting-state fMRI was acquired using multiband gradient echo-planar imaging with multiband factor 8, TR=720 ms, TE=37 ms, 2 mm isotropic voxels, 15 min duration, and 1,238 volumes^53^; high-resolution T1-weighted and T2-weighted structural images were acquired using 0.8 mm isotropic 3D MP-RAGE^54^ and Siemens SPACE^55^ sequences, respectively. In the Replication cohort, resting-state fMRI was acquired using single-shot gradient echo-planar imaging with TR=1500 ms, TE=30 ms, 3 mm in-plane resolution, 4 mm slice thickness, 15 min duration, and 600 volumes; structural images were acquired using 1 mm isotropic T1-weighted MPRAGE and T2-weighted spin-echo sequences.

HCP-YA imaging was acquired on a customized 3.0 T Siemens Skyra scanner using the standardized WU-Minn HCP acquisition protocol^53,56^. Diffusion MRI was acquired at 1.25 mm isotropic resolution using a multi-shell spin-echo echo-planar protocol with TR=5,520 ms and TE=89.5 ms. The protocol included 270 diffusion-weighted gradient directions distributed across three nominal b-value shells of approximately 1000, 2000, and 3000 s/mm^2^, with 90 directions per shell, interspersed low-b-value images, and reversed phase-encoding acquisitions for susceptibility-distortion correction.

### MRI preprocessing

Diffusion MRI data were preprocessed using QSIPrep^57^ 1.0.2, built on Nipype^58^ 1.9.1. The anatomical reference was reoriented to AC-PC alignment and nonlinearly registered to the MNI152NLin2009cAsym template using ANTs^59^. T1-weighted brain extraction and segmentation were performed using SynthStrip^60^ and SynthSeg^61^, respectively. Diffusion images were grouped by phase-encoding polarity, denoised with MRtrix3^62^ dwidenoise, bias-corrected with MRtrix3 dwibiascorrect, and corrected for susceptibility related distortion, eddy currents, and head motion using FSL^63^ TOPUP^64^ and EDDY^65^. Volumes with b-values<100 s/mm^2^ were treated as b0 images, and b0 intensities were normalized across DWI series. The corrected DWI data were resampled to AC-PC space at 2 mm isotropic resolution, and motion and quality-control confounds, including framewise displacement and slice-wise cross-correlation, were generated. Tensor-derived scalar maps were subsequently generated from the corrected AC-PC diffusion data using the QSIrecon^57^ DSI Studio scalar workflow. Fractional anisotropy (FA) and mean diffusivity (MD) maps were exported in AC-PC space for each subject and session and used for cerebellar microstructural analyses.

Resting-state fMRI data were preprocessed using the Human Connectome Project minimal preprocessing pipeline^66^. Structural images were processed with FreeSurfer^67^ v5.3, and functional preprocessing included gradient distortion correction, motion correction, field map-based distortion correction, brain masking, and normalization to 2 mm isotropic MNI space using FSL v5.0. White matter and cerebrospinal fluid signals, together with six rigid-body motion parameters, were regressed from the functional time series, which were then detrended and bandpass filtered between 0.01 and 0.1 Hz. Surface-based structural and functional data were represented in 32k CIFTI space, comprising 32,492 cortical vertices per hemisphere and 91,282 total grayordinates across cortex and subcortex. Preprocessing quality was assessed by visual inspection of head motion, signal-to-noise ratio, and registration accuracy.

### Brain parcellation

Brain parcellation was performed using the Integrated Cortico-Subcortical-Cerebellar Atlas^31^ (iCSC-438) in CIFTI space. The atlas combines the 360 cortical parcels of the multimodal human cortical parcellation^68^, 50 thalamic and basal ganglia parcels from the Melbourne Subcortex Atlas^69^, and 28 cerebellar parcels from a probabilistic atlas of the human cerebellum^70^. For each scan, parcel-wise functional connectomes were computed as 438 × 438 correlation matrices using Connectome Workbench (https://www.humanconnectome.org/software/get-connectome-workbench). For region-level analyses, the iCSC438 atlas was transformed from MNI152NLin2009cAsym space to each participant’s AC-PC space using the corresponding QSIPrep MNI-to-ACPC transform and resampled with nearest-neighbor interpolation to preserve parcel labels.

### Structural Connectivity Gradient Mapping

We developed Structural Connectivity Gradient Mapping (SCGM) to derive low-dimensional representations of whole-brain structural connectivity from parcelwise diffusion MRI connectomes. SCGM integrates structural-connectivity estimation, affinity construction, nonlinear embedding, gradient alignment, and reproducibility assessment into a unified framework for structural connectome gradient mapping.

Structural connectomes were reconstructed using QSIRecon^57^ 1.1.2, built on Nipype 1.9.1. Atlas parcellations were transformed from template space into diffusion space using T1-weighted spatial normalization from QSIPrep. Whole-brain tractography was performed using the QSIRecon MRtrix3 reconstruction workflow. Multi-tissue response functions were estimated using the Dhollander algorithm, and fiber orientation distributions were estimated using multi-tissue constrained spherical deconvolution. FODs were intensity-normalized using mtnormalize. Probabilistic tractography was performed with MRtrix3 tckgen using the iFOD2 algorithm and anatomically constrained tractography based on a T1-weighted five-tissue-type segmentation, including gray matter–white matter interface seeding and cropping, backtracking, and anatomically informed streamline termination. Streamline weights were estimated using spherical-deconvolution informed filtering of tractograms 2 (SIFT2^30^) and used to construct subject-level structural connectivity matrices in iCSC-438 space.

Primary structural connectomes were reconstructed using an FOD amplitude cutoff of 0.10 during streamline propagation. To assess robustness to tractography thresholding, we reconstructed a second set of connectomes using a more permissive FOD cutoff of 0.03. The lower cutoff allowed tracking to continue through lower-amplitude FOD compartments and was used as a sensitivity analysis for long-range, low-amplitude, or geometrically complex pathways. Primary analyses were based on FOD=0.10 connectomes, and FOD=0.03 analyses were used to assess robustness. To reduce distance- and parcel-size-related biases in structural connectivity weights, we generated residualized structural connectomes. For each subject-session scan, raw SIFT2-weighted structural connectivity values were regressed on mean streamline length and parcel-size terms. Mean streamline length was computed as the average physical length, in millimeters, of streamlines assigned to each parcel pair. Parcel-size terms were derived from the sizes of the two connected parcels. Residuals from this model were retained as length- and size-adjusted structural connectivity weights and used for structural-gradient analyses.

Structural connectivity gradients were estimated using diffusion-map embedding^23^ of parcel-wise structural connectivity profiles. For each subject-session, the residualized iCSC-438 structural connectivity matrix was symmetrized, and each parcel was represented by its structural connectivity profile. Pairwise cosine similarity between parcel connectivity profiles was computed and linearly rescaled to form a non-negative affinity matrix:

$$A_{\text{ij}}=\frac{1+\text{cos}\left({\text{w}}_{i},{\text{w}}_{j}\right)}{2},$$

where **w**_*i*_ and **w**_*j*_ are the structural connectivity profiles of parcels *i* and *j*. Diffusion-map embedding^23^ was then applied to the affinity matrix using α=0.5, generating 50 structural connectivity gradients per scan with mapalign Python package (https://github.com/sensein/mapalign). Gradients were expanded back to the full 438-parcel atlas space and saved in CIFTI format for subsequent group-level and spatial-alignment analyses. To enable comparison across participants and scans, individual gradients were aligned to group-level template gradients generated from the average parcellated structural connectome using the same procedure.

### Functional connectivity gradient estimation

Functional connectivity gradients^4^ were estimated from parcel-wise functional connectomes^31,71^. For each scan, a 438 × 438 Pearson correlation matrix was computed using the iCSC-438 atlas. The strongest 10% of positive connections in each row were retained to generate a sparse adjacency matrix, and cosine similarity between regional connectivity profiles was computed to construct the affinity matrix. Diffusion map embedding with α=0.5 was applied with mapalign, yielding 50 functional connectivity gradients per scan. Individual gradients were aligned to group-level template of the functional gradients generated from the average parcellated functional connectome. Specifically, for each scan, the components with the strongest spatial correlations with the template were identified, sign-aligned to the corresponding template gradients, and carried forward for subsequent analyses.

### Endpoint definitions and tract-loading measures

To characterize the anatomical axes underlying higher-order structural gradients, we defined cortical endpoint sets corresponding to putative tract or system-level contrasts (Table S1). For G2, endpoint parcels were assigned to dorsal or ventral association systems, reflecting superior longitudinal/arcuate versus inferior longitudinal/inferior fronto-occipital/uncinate pathways. For G3, endpoint parcels were assigned to cingulate or temporal systems. For G4, endpoint parcels were assigned to cerebellar or transmodal systems, with transmodal endpoints comprising default-mode, frontoparietal/control, and lateral temporal association territories.

For each anatomical contrast, we computed parcel-wise endpoint-set-specific weighted degree from the group-mean structural connectome. For endpoint set *E*, the size-normalized partial weighted degree of parcel *i* with respect to was defined as:

$$L_{i}\left(E\right)=\frac{1}{\left|E\right|}\sum_{j\in E}w_{\text{ij}},$$

where *w*_*ij*_ denotes the structural connectivity weight between parcels *i* and *j*. Loadings were computed for dorsal, ventral, cingulate, temporal, cerebellar, and transmodal endpoint sets. Signed contrasts were defined as dorsal-ventral, cingulate-temporal, and cerebellar-transmodal loading axes. Positive values indicated relatively stronger size-normalized connectivity to dorsal, cingulate, or transmodal endpoint sets, whereas negative values indicated relatively stronger connectivity to ventral, temporal, or cerebellar endpoint sets. These contrasts were used in nested regression models to test whether structural gradients reflected anatomical endpoint axes beyond Cartesian parcel geometry.

### Anatomical models of structural gradients

To assess whether structural gradients reflected gross spatial geometry or tract-defined anatomical organization, we used nested parcel-wise ordinary least-squares models. For each parcel, gradient values were modeled as a function of Cartesian centroid coordinates, anatomical loading contrast, or both. Geometry-only models tested whether a gradient could be explained by smooth spatial variation across the brain. Combined models tested whether tract-derived loading contrasts explained additional variance beyond geometry. For G2–G4, the anatomical contrasts were the dorsal-ventral, cingulate-temporal, and cerebellar-transmodal loading axes, respectively. For G1, which was dominated by laterality, hemisphere was substituted for the tract-loading predictor, and coordinate-by-hemisphere interaction models were used to test whether the spatial expression of G1 differed across hemispheres. Models were compared using R^2^, adjusted R^2^, Akaike information criterion, Bayesian information criterion, and nested-model tests. Continuous predictors were standardized before model fitting. For subject-level analyses, the same models were fit separately for each subject-session scan, and distributions of model fit, ΔR^2^, and coefficients were summarized across scans.

### Structure–function gradient correspondence

Spatial correspondence between structural and functional gradients was assessed across the 360 cortical parcels of the iCSC-438 atlas. Gradients were matched by maximal spatial correspondence and sign-aligned before comparison. Spatial correspondence was quantified using Pearson and Spearman correlations. Geometry-independent correspondence was assessed by separately residualizing structural and functional gradients with respect to Cartesian parcel coordinates and correlating the residuals. We also used nested parcel-wise linear models to test whether structural gradients explained functional-gradient topography beyond geometry:

$${\text{FC}}_{i}=\beta_{0}+\beta_{x}x_{i}+\beta_{y}y_{i}+\beta_{z}z_{i}+\beta_{\text{SC}}{\text{SC}}_{i}+\epsilon_{i}.$$

Combined models were compared with geometry-only and SC-only models using R^2^, adjusted R^2^, Akaike information criterion, Bayesian information criterion, and ΔR^2^. Spatial-autocorrelation-preserving significance was assessed using hemisphere-constrained spin tests with 10,000 rotations. Spin-based p values were calculated from the fraction of rotated null maps with correlations ≥ the observed correlation.

### Demographic effects

Age and sex effects were tested using parcel-wise linear mixed-effects models after component matching and sign alignment to the corresponding group-level template. Models were fit separately for each modality, gradient, and parcel. Aligned gradient values were modeled as a function of age, sex, modality-specific covariates, and a participant-level random intercept to account for repeated sessions. Sex was coded as a binary indicator with female as the reference group. For structural-gradient models, covariates included intracranial volume and mean cortical thickness estimated from FreeSurfer. For functional-gradient models, covariates included mean framewise displacement computed from rigid-body motion regressors. Age, intracranial volume, cortical thickness, and mean framewise displacement were z-scored before modeling.

### Cerebellar-transmodal graph analyses

To further characterize the G4 cerebellar-transmodal axis, graph-theoretic analyses were performed using non-residualized MRtrix structural connectivity matrices from the primary FOD=0.10 reconstruction. Weighted iCSC-438 connectivity matrices were treated as undirected weighted graphs, restricted to non-negative edge weights, and diagonal entries were removed. Cerebellar/G4-negative-pole parcels and transmodal parcels were defined from the iCSC-438 parcel labels and endpoint annotations used in the G4 anatomical model.

Local segregation of the cerebellar parcel set was quantified using the mean and median weighted clustering coefficient^72^. Specifically, cerebellar-transmodal integration was quantified using two complementary measures: median shortest-path graph distance from cerebellar/G4-negative-pole parcels to transmodal parcels, after converting connection weights to edge lengths using an inverse-weight transform; and median direct structural connectivity weight between cerebellar/G4-negative-pole parcels and transmodal parcels. Age effects on each graph metric were tested using linear mixed-effects models adjusted for sex and intracranial volume, with participant-specific random intercepts to account for repeated sessions.

Multiple comparisons were controlled using Benjamini-Hochberg false-discovery-rate correction within each gradient and across all tested gradient-parcel combinations, as specified for each analysis. Statistical analyses were implemented in Python 3.10.

## Supplementary Material

Supplementary Files

This is a list of supplementary files associated with this preprint. Click to download.

- Supplementary5.pdf

## Figures and Tables

**Figure 1.**
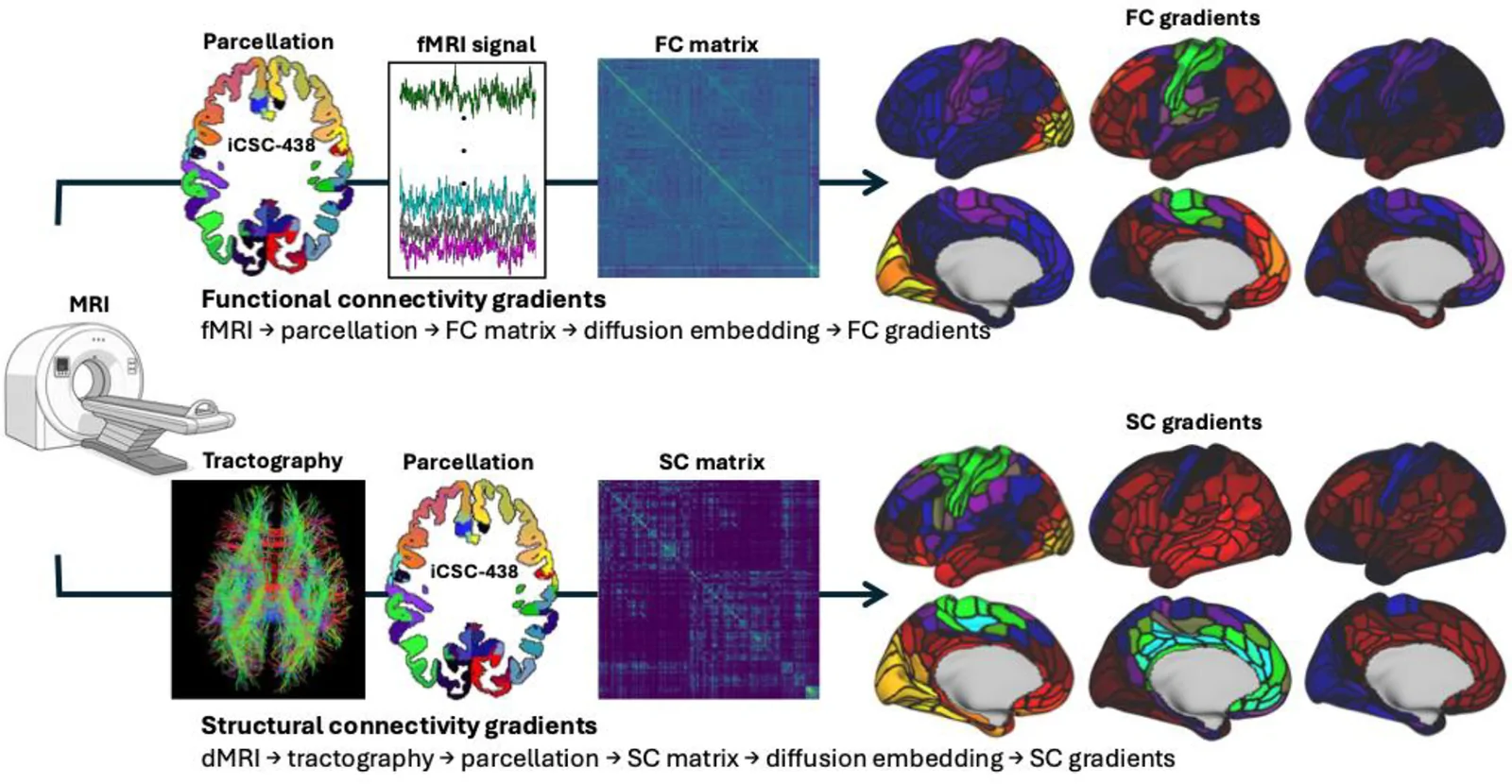
Overview of functional and structural gradient construction. Functional gradients were estimated from resting-state functional connectivity matrices, whereas structural gradients were estimated from SIFT2-weighted tractography-derived structural connectivity matrices. Both modalities were represented in the iCSC-438 parcellation and embedded using diffusion map embedding to identify low-dimensional axes of cortical connectivity organization.

**Figure 2.**
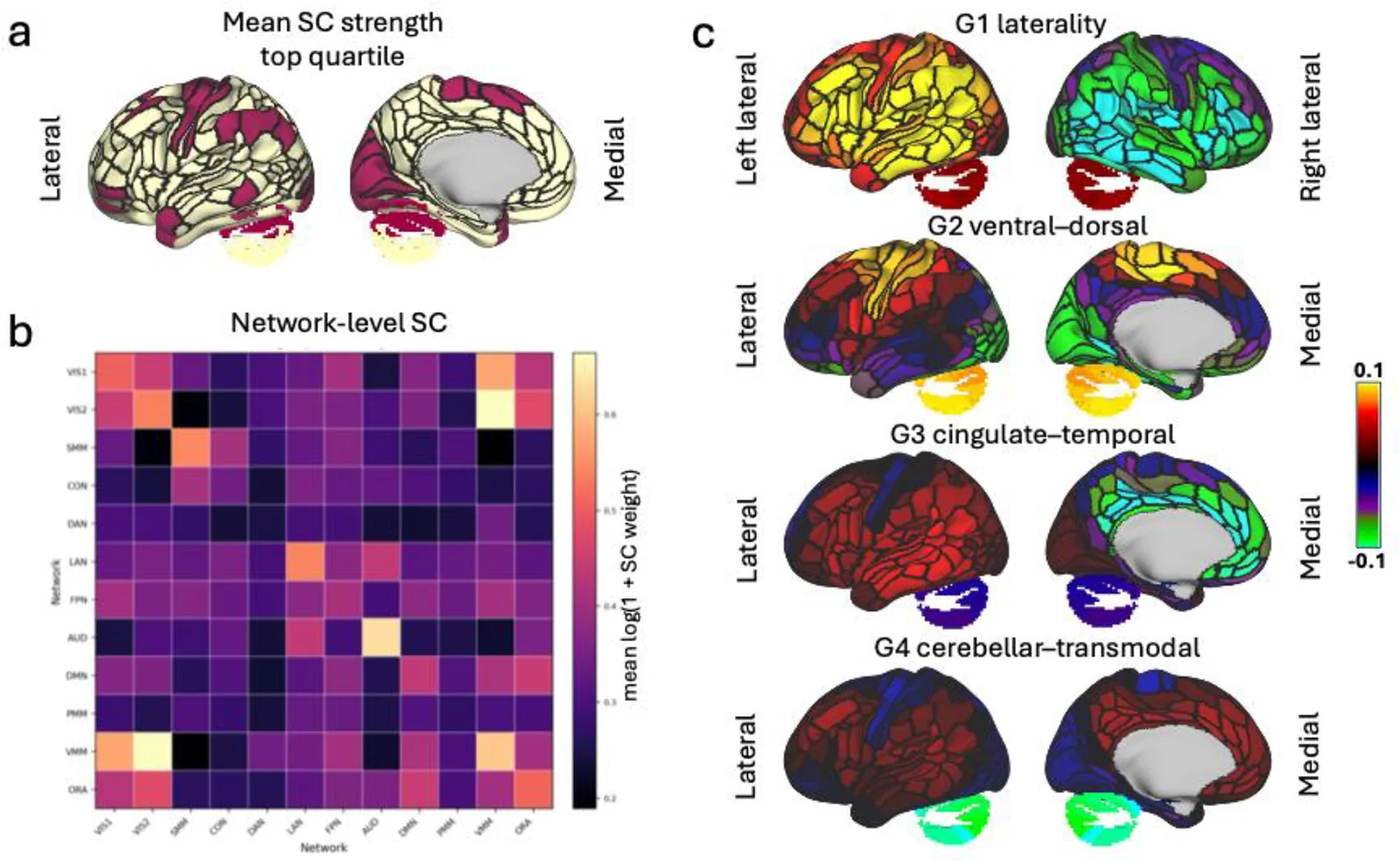
Group-mean SC organization and gradient topographies. **a**, Cortical and cerebellar distributions of high mean SC-strength parcels, defined descriptively as parcels in the upper quartile of the parcel-wise mean weighted connectivity distribution. **b**, Schematic network-level summary of the group-mean structural connectome, computed by averaging log-transformed structural connectivity weights between iCSC-438 network assignments. **c**, Surface and cerebellar maps of the first four group-level SC gradients. For G1, left and right lateral views are shown to emphasize the dominant laterality axis; for G2-G4, lateral and medial views are shown to highlight dorsal-ventral, cingulate-temporal, and cerebellar-transmodal topographies, respectively. Discovery cohort (n=61). Network assignment^38^: Visual (VIS1, VIS2), Somatomotor (SMM), Cingulo-Opercular (CON), Dorsal Attention (DAN), Language (LAN), Frontoparietal (FPN), Auditory (AUD), Default-Mode (DMN), Posterior Multimodal (PMM), Ventral Multimodal (VMM), and Orbito-Affective (ORA).

**Figure 3.**
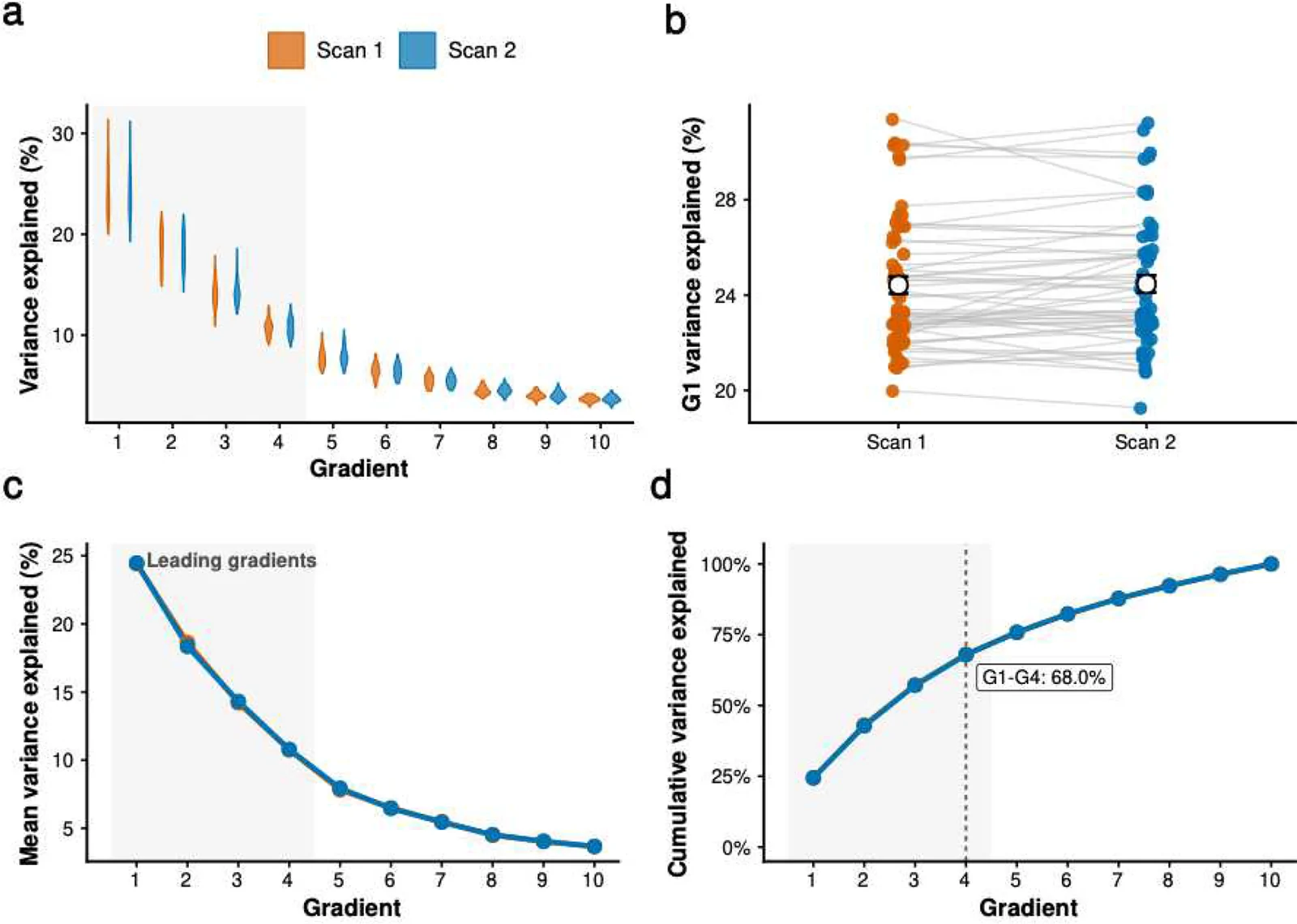
Variance explained by structural connectivity gradients across scans. **a**, Distribution of relative variance explained by the first 10 gradient components across subjects for scan 1 and scan 2. Violin plots show the subject-level distributions, with points representing individual subjects. The first few components explain the largest proportion of variance, followed by a gradual decrease across higher-order components. **b**, Subject-level paired comparison of variance explained by the first gradient component across scans. Lines connect the same subject across scan 1 and scan 2, illustrating within-subject scan-to-scan stability. **c**, Mean relative variance explained by each of the first 10 gradient components for scan 1 and scan 2. Points show the across-subject mean for each component, and error bars/ribbons indicate the standard error of the mean where shown. **d**, Cumulative relative variance explained by the first 10 gradient components. The curve shows the cumulative contribution of components 1 through 10, demonstrating that most variance is captured by the leading gradient components, with progressively smaller gains from higher-order components. Discovery cohort (n=61).

**Figure 4.**
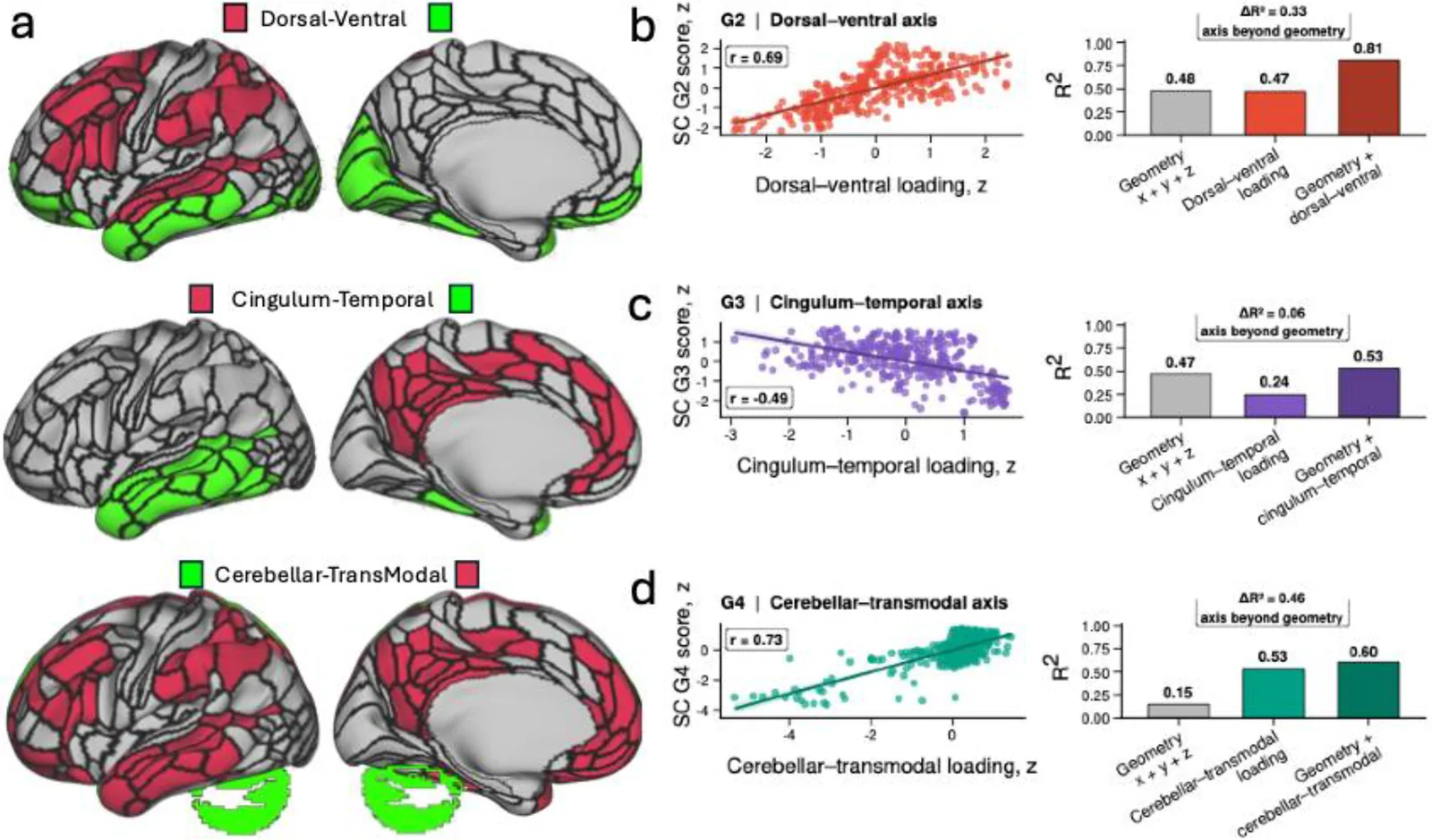
Tract-derived anatomical interpretation of SC gradients. **a**, Endpoint sets used to define tract-loading contrasts for G2-G4. Each row shows the two opposing endpoint classes used to compute a signed parcel-wise loading contrast. **b-d**, Parcel-wise associations between standardized SC gradient scores and standardized tract-loading contrasts. Adjacent bar plots show variance explained by Cartesian geometry, the tract-loading contrast, and combined models. For G4, an additional model with cerebellar and transmodal loadings entered separately is shown. Discovery cohort (n=61).

**Figure 5.**
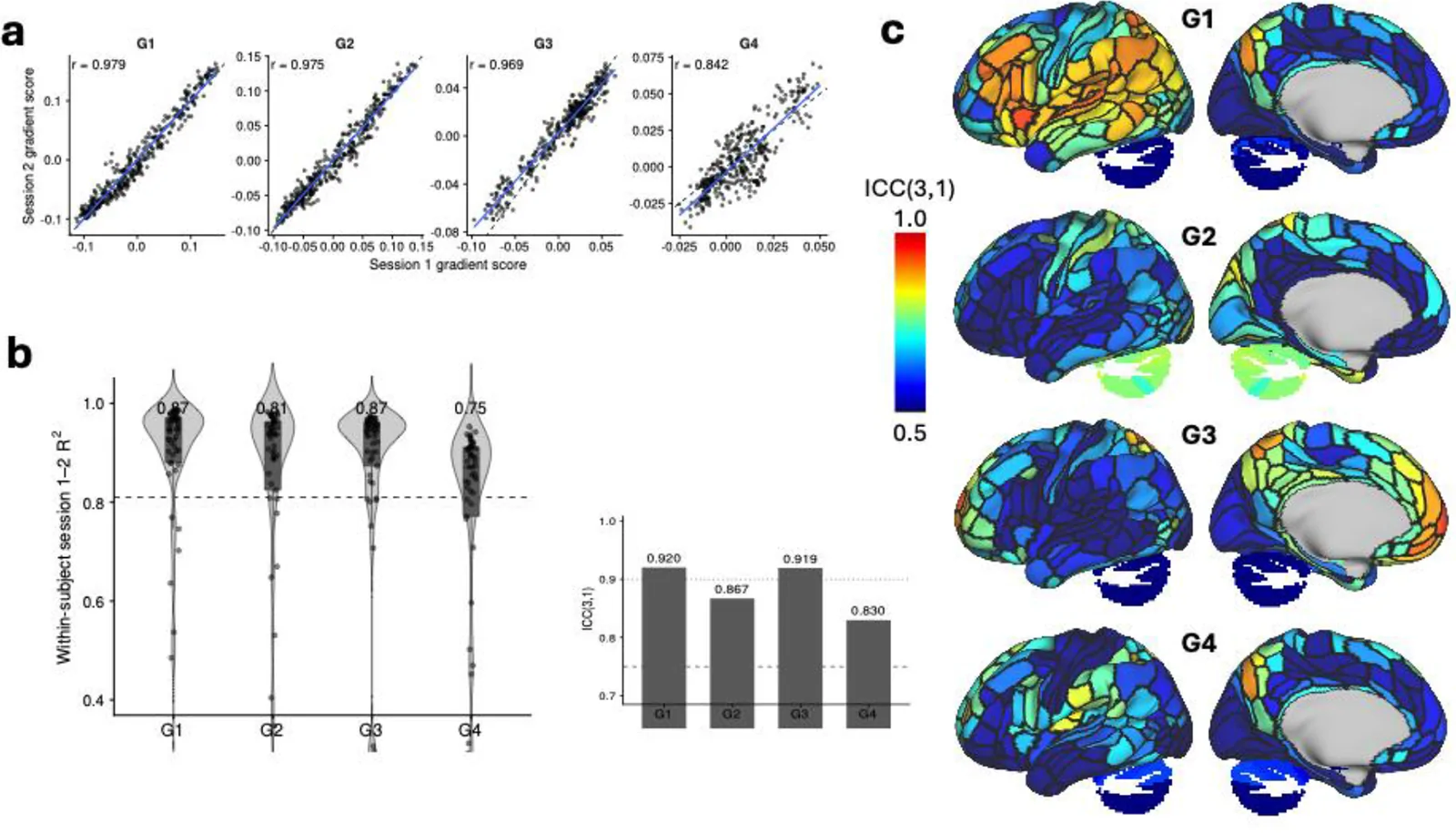
Test-retest reliability of structural connectivity gradients. **a**, Example test-retest reproducibility in a representative participant. Scatterplots show parcel-wise correspondence between session 1 and session 2 aligned SC gradient scores for G1-G4. Each point represents a cortical parcel; blue lines indicate linear fits. **b**, Subject-level test-retest reproducibility. Violin and box plots show within-participant session 1-session 2 spatial correlations across cortical parcels; points denote individual participants. **c**, Parcel-wise ICC(3,1) maps for aligned SC gradients, showing the spatial distribution of reliability across the brain. The inset summarizes mean ICC(3,1) values for each gradient. Overall, SC gradients showed high test-retest reliability, with strongest reproducibility for G1-G3 and moderately lower reliability for G4. Discovery cohort (n=61).

**Figure 6.**
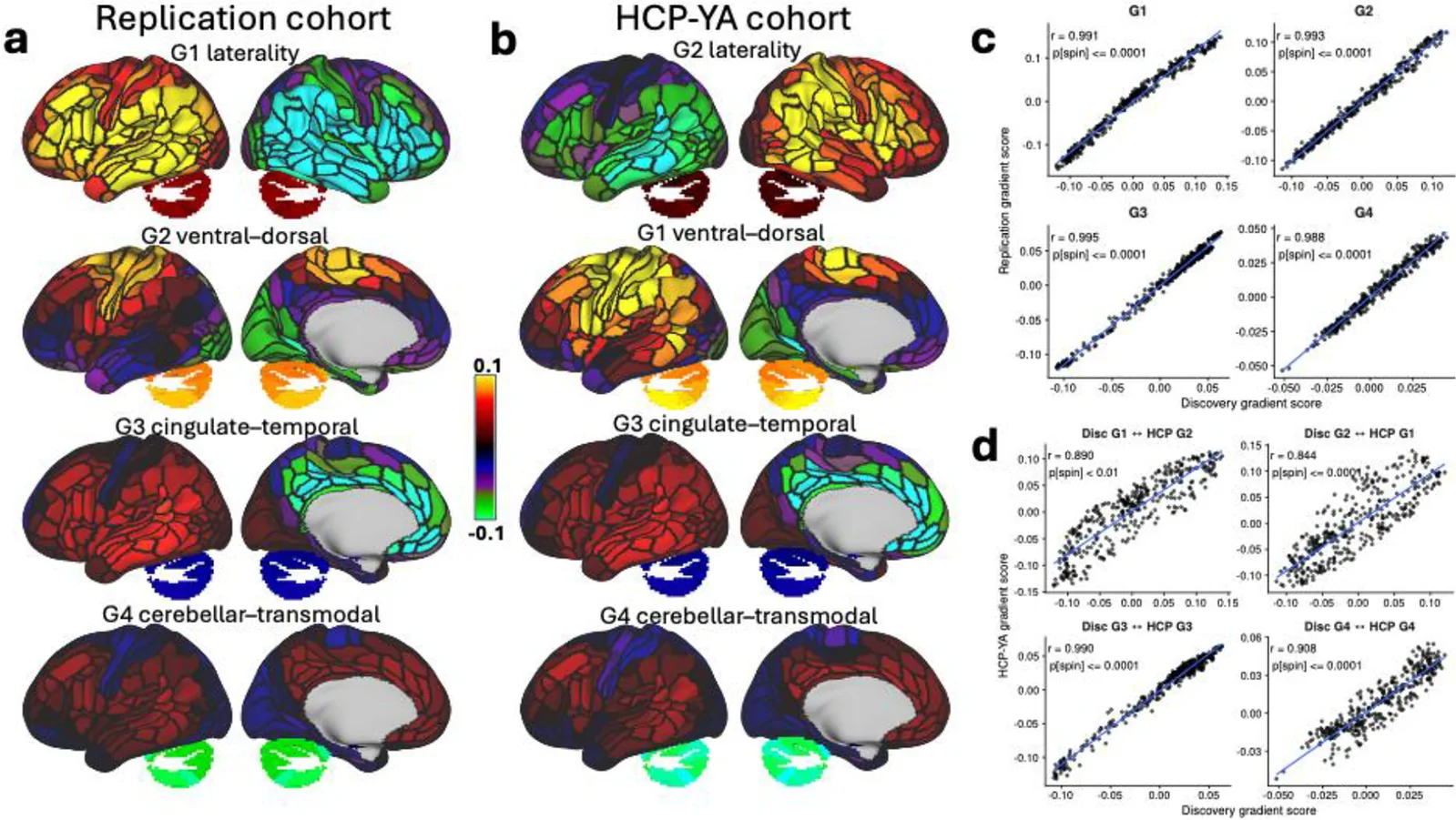
Out-of-sample reproducibility and external validation of SC gradients. **a**, Group-level SC gradients estimated in the onsite Replication cohort (n=28) acquired using a different RF coil. Maps are shown after component matching and sign alignment to the Discovery reference gradients. **b**, Group-level SC gradients estimated in the external HCP-YA cohort (n=100), also shown after component matching and sign alignment to the Discovery reference. In HCP-YA, the first two components were reordered relative to Discovery, such that Discovery G1 matched HCP-YA G2 and Discovery G2 matched HCP-YA G1, whereas G3 and G4 matched directly. **c**, Parcel-wise Discovery-Replication correspondence for matched SC gradients across cortical parcels. **d**, Parcel-wise Discovery-HCP-YA correspondence for matched SC gradients across cortical parcels. Each point represents a cortical parcel; blue lines indicate linear fits. High correlations across onsite and external validation datasets demonstrate robust recovery of the SC gradient architecture beyond the Discovery cohort (n=61).

**Figure 7.**
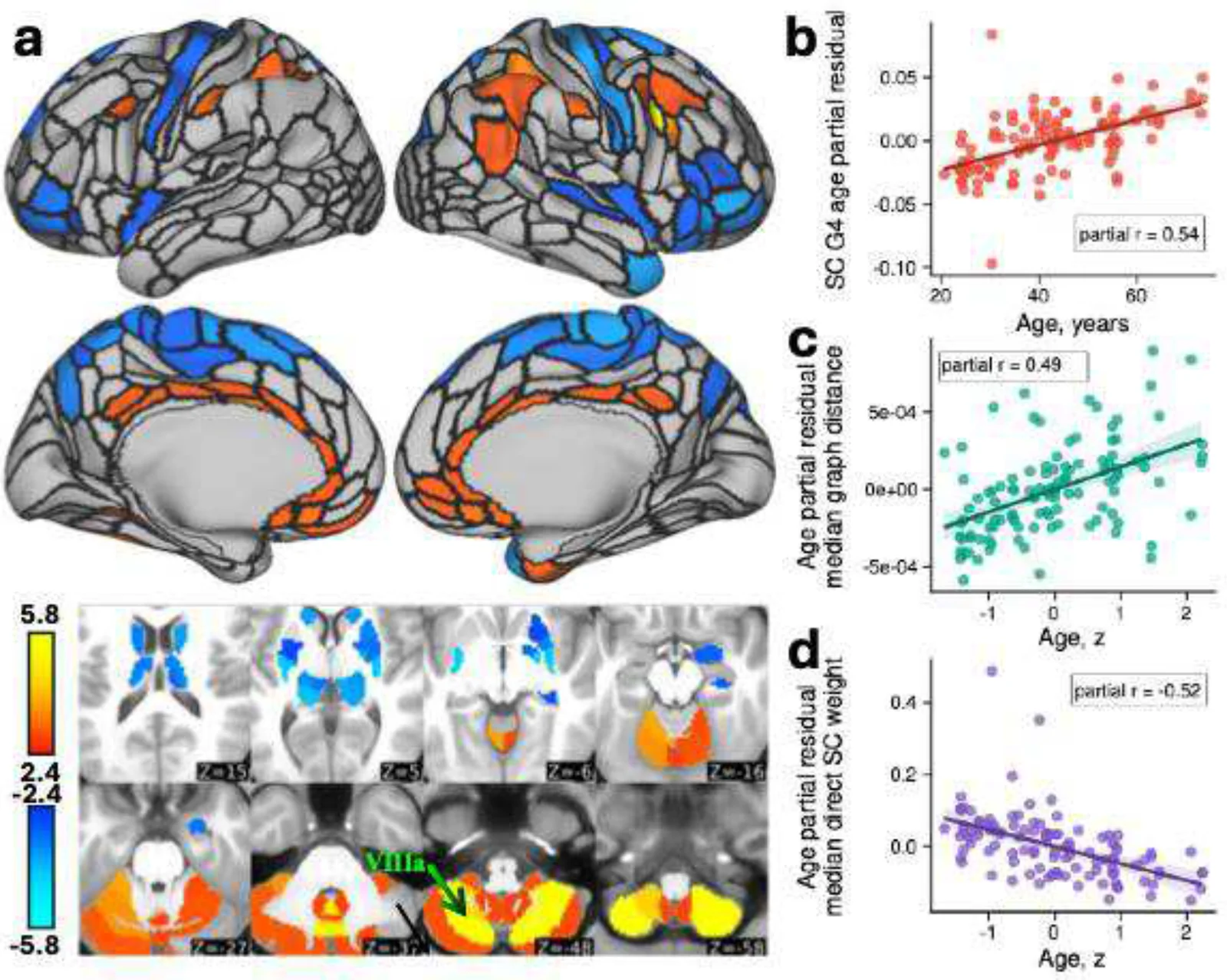
Age-related variation in the structural cerebellar-transmodal gradient. **a**, Parcel-wise age effects on SC G4 from linear mixed-effects models adjusted for sex and intracranial volume, with participant-specific random intercepts. Maps show FDR-thresholded fixed-effect statistics for age across cortical, subcortical, and cerebellar parcels. Warm colors indicate positive age associations and cool colors indicate negative age associations. Positive age effects were most prominent in the cerebellar negative pole of G4, consistent with age-related attenuation of this pole. **b**, Partial-residual age association for the strongest SC G4 age-effect parcel (VIIIa), illustrating the fitted age effect after accounting for sex, intracranial volume, and participant-specific intercepts. **c-d**, Graph-level analyses of structural connectivity showed that older age was associated with increased median graph distance from G4 cerebellar to transmodal parcels (**c**), with a directionally consistent reduction in median direct cerebellar-transmodal connectivity weight (**d**). Discovery cohort (n=61; age=43.3±13.7 years; 34 females). Tractography performed with an FOD amplitude threshold of 0.10.

## Data Availability

ROI summary data generated in this study will be made available in Figshare. Deidentified individual-level images can be obtained from the corresponding author upon reasonable request. Per NIH policy, sharing requires Data Transfer Agreements between institutions to ensure compliance with privacy and institutional requirements.

## Code availability

The Structural Connectivity Gradient Mapping (SCGM) pipeline and code used to reproduce the principal analyses reported in this study will be made publicly available through GitHub upon publication. During peer review, the code will be made available to editors and reviewers upon request.
